# Disease severity-based subgrouping of type 2 diabetes does not parallel differences in quality of life: the Maastricht Study

**DOI:** 10.1007/s00125-023-06082-4

**Published:** 2024-01-11

**Authors:** Nikki C. C. Werkman, Gema García-Sáez, Johannes T. H. Nielen, Jose Tapia-Galisteo, Francisco J. Somolinos-Simón, Maria E. Hernando, Junfeng Wang, Li Jiu, Wim G. Goettsch, Carla J. H. van der Kallen, Annemarie Koster, Casper G. Schalkwijk, Hein de Vries, Nanne K. de Vries, Simone J. P. M. Eussen, Johanna H. M. Driessen, Coen D. A. Stehouwer

**Affiliations:** 1 Cardiovascular Research Institute Maastricht (CARIM), Maastricht University, Maastricht, the Netherlands; 2 Department of Clinical Pharmacy and Toxicology, Maastricht University Medical Center+, Maastricht, the Netherlands; 3 Bioengineering and Telemedicine Group, Centro de Tecnología Biomédica, ETSI de Telecomunicación, Universidad Politécnica de Madrid, Madrid, Spain; 4 Centro de Investigación Biomédica en Red (CIBER)-BBN: Networking Research Center for Bioengineering, Biomaterials and Nanomedicine, Madrid, Spain; 5 Division of Pharmacoepidemiology and Clinical Pharmacology, Utrecht Institute for Pharmaceutical Sciences, Utrecht University, Utrecht, the Netherlands; 6 National Health Care Institute, Diemen, the Netherlands; 7 Department of Internal Medicine, Maastricht University Medical Center+, Maastricht, the Netherlands; 8 Department of Social Medicine, Maastricht University, Maastricht, the Netherlands; 9 School for Public Health and Primary Care (CAPHRI), Maastricht University, Maastricht, the Netherlands; 10 Department of Epidemiology, Maastricht University, Maastricht, the Netherlands

**Keywords:** Complications, Quality of life, Subgroups

## Abstract

**Aims/hypothesis:**

Type 2 diabetes is a highly heterogeneous disease for which new subgroups (‘clusters’) have been proposed based on disease severity: moderate age-related diabetes (MARD), moderate obesity-related diabetes (MOD), severe insulin-deficient diabetes (SIDD) and severe insulin-resistant diabetes (SIRD). It is unknown how disease severity is reflected in terms of quality of life in these clusters. Therefore, we aimed to investigate the cluster characteristics and cluster-wise evolution of quality of life in the previously defined clusters of type 2 diabetes.

**Methods:**

We included individuals with type 2 diabetes from the Maastricht Study, who were allocated to clusters based on a nearest centroid approach. We used logistic regression to evaluate the cluster-wise association with diabetes-related complications. We plotted the evolution of HbA_1c_ levels over time and used Kaplan–Meier curves and Cox regression to evaluate the cluster-wise time to reach adequate glycaemic control. Quality of life based on the Short Form 36 (SF-36) was also plotted over time and adjusted for age and sex using generalised estimating equations. The follow-up time was 7 years. Analyses were performed separately for people with newly diagnosed and already diagnosed type 2 diabetes.

**Results:**

We included 127 newly diagnosed and 585 already diagnosed individuals. Already diagnosed people in the SIDD cluster were less likely to reach glycaemic control than people in the other clusters, with an HR compared with MARD of 0.31 (95% CI 0.22, 0.43). There were few differences in the mental component score of the SF-36 in both newly and already diagnosed individuals. In both groups, the MARD cluster had a higher physical component score of the SF-36 than the other clusters, and the MOD cluster scored similarly to the SIDD and SIRD clusters.

**Conclusions/interpretation:**

Disease severity suggested by the clusters of type 2 diabetes is not entirely reflected in quality of life. In particular, the MOD cluster does not appear to be moderate in terms of quality of life. Use of the suggested cluster names in practice should be carefully considered, as the non-neutral nomenclature may affect disease perception in individuals with type 2 diabetes and their healthcare providers.

**Graphical Abstract:**

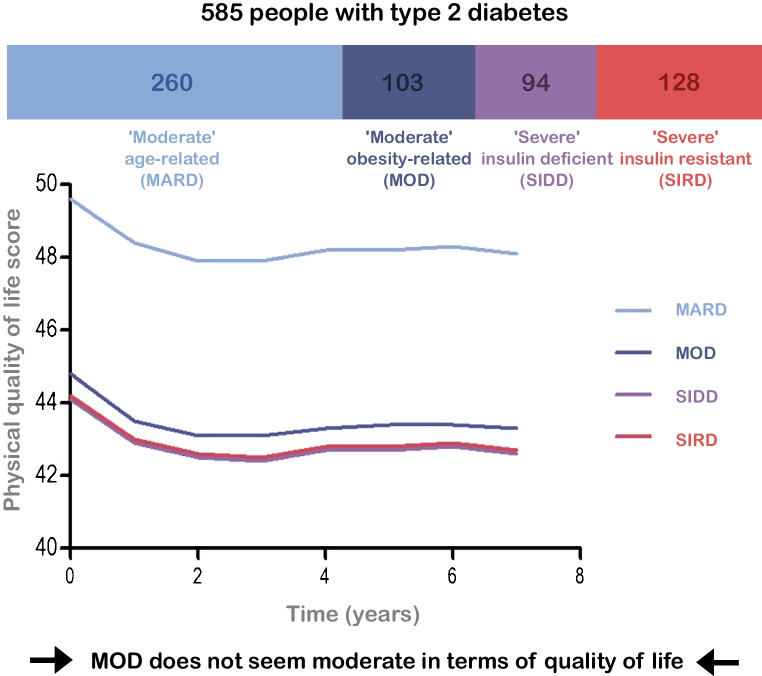

**Supplementary Information:**

The online version of this article (10.1007/s00125-023-06082-4) contains peer-reviewed but unedited supplementary material.


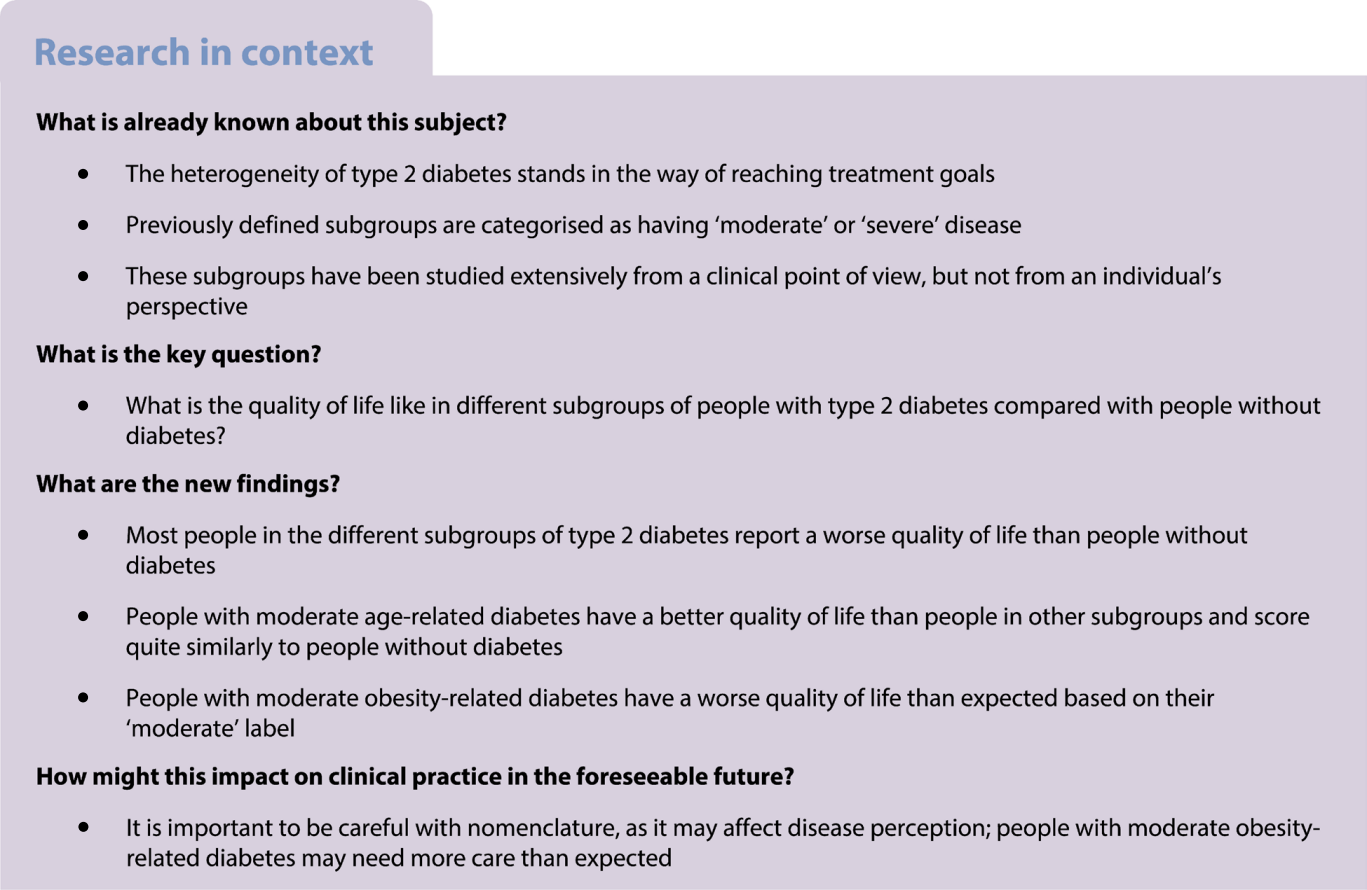


## Introduction

Type 2 diabetes is a highly heterogeneous disease; almost half a billion people [1] living with this condition present with various profiles of physical characteristics, metabolic function and disease severity [2]. As a result, a large group of highly different people are classified as having the same condition. Consequently, this makes it difficult to meet their needs in terms of appropriate care for every individual.

In 2018, Ahlqvist et al identified four subgroups of people with type 2 diabetes based on disease severity and metabolic variables: moderate age-related diabetes (MARD), moderate obesity-related diabetes (MOD), severe insulin-deficient diabetes (SIDD) and severe insulin-resistant diabetes (SIRD) [3]. Subsequently, several studies have replicated the same subgroups using a clustering algorithm [4–9], and others have used a nearest centroid approach to allocate individuals to the proposed subgroups [10, 11]. Evaluation of subgroup characteristics showed that SIDD was the most therapy-resistant subgroup [9] and SIRD was the subgroup with the lowest physical fitness level [10] and the highest risk of chronic kidney disease (CKD) [3, 5, 6].

Although studies have replicated the subgroups and have shown important differences between them, these subgroups have not yet been implemented in clinical practice. Interestingly, the names given to the subgroups incorporate a qualitative description of disease severity or degree of metabolic derangement: two ‘moderate’ subgroups and two ‘severe’ subgroups. This disease-related nomenclature is not neutral, even though the subgroups are based on few (clinical) variables. However, it is unknown whether the moderate and severe states are reflected in individuals’ everyday well-being. As patient views are known to differ from those which doctors perceive as important [12, 13], and diabetes has been associated with impaired quality of life (QoL) [14, 15], it would also be relevant to study how the ‘moderate’ and ‘severe’ stages of the clusters are reflected in individuals’ QoL.

Therefore, we aimed to investigate the evolution of QoL in people in each cluster of type 2 diabetes compared with people without diabetes.

## Methods

### Data source

We used data from the Maastricht Study, an observational, prospective, population-based cohort study. The rationale and methodology have been described previously [16]. In brief, the study focuses on the aetiology, pathophysiology, complications and comorbidities of type 2 diabetes and is characterised by an extensive phenotyping approach. All individuals between 40 and 75 years of age living in the southern part of the Netherlands were eligible for participation. Participants were recruited through mass media campaigns, from municipal registries and from the regional Diabetes Patient Registry via mailings. Recruitment was stratified according to known type 2 diabetes status, with an oversampling of individuals with type 2 diabetes for reasons of efficiency. The present study included the first participants, who completed the baseline survey between November 2010 and November 2013. We used data until 2013 only, as some of the required variables, such as homeostatic model assessments, are not yet available in the newer data. The examinations of each participant were performed within a time window of 3 months after the baseline visit. The Maastricht Study has the approval of the institutional medical ethics committee (NL31329.068.10) and the Dutch Ministry of Health, Welfare and Sport (Permit 131088-105234-PG). All participants gave their written informed consent. How representative the study sample is of the source population in the study region is monitored continuously as described elsewhere [16].

The current study is part of the HTx Project. HTx is a Horizon 2020 project supported by the European Union and lasting for 5 years from January 2019. The main aim of HTx is to create a framework for the Next Generation Health Technology Assessment to support patient-centred, societally oriented, real-time decision-making on access to and reimbursement for health technologies throughout Europe.

### Study population

From the Maastricht Study dataset, we selected all people with type 2 diabetes based on the OGTT performed during their first (baseline) visit to the study centre or use of glucose-lowering drugs based on the WHO definition [17]. Type 2 diabetes was defined by a fasting glucose level ≥7.0 mmol/l and 2 h post-glucose drink glucose level ≥11.1 mmol/l, or the use of glucose-lowering drugs, and the absence of a type 1 diabetes diagnosis. We excluded individuals with missing values in the variables needed for clustering and subsequently those with outliers (>3 SD from the mean) in these variables. People were allocated to the newly diagnosed group if they had never been diagnosed with diabetes before (based on a baseline questionnaire) and did not use medication for diabetes at baseline but were classified as having diabetes according to the OGTT at baseline. The other people were included in the already diagnosed group. Separately, we included people without diabetes from the Maastricht Study dataset, according to the baseline OGTT (fasting glucose level <6.1 mmol/l and 2 h post-glucose drink glucose level <7.8 mmol/l) and no use of glucose-lowering drugs, in order to plot their QoL for comparison with people with diabetes. People with prediabetes (fasting glucose level <7.0 mmol/l and 2 h post-glucose drink glucose level <11.1 mmol/l, and no use of glucose-lowering drugs) were excluded. Sex was self-reported and the Maastricht Study only provided the options ‘male’ and ‘female’. The definitions used are according to the Maastricht Study methodology [16].

### Clustering

Individuals were assigned to clusters using the nearest centroid approach using the centroids published by Ahlqvist et al [3]. They identified clusters through a data-driven cluster analysis using *k*-means and hierarchical clustering in individuals with newly diagnosed diabetes from a Swedish cohort. The clustering variables for type 2 diabetes included age at diagnosis, BMI, HbA_1c_, HOMA-B and HOMA-IR (using HOMA2 and C-peptide levels). The resulting clusters were MARD, MOD, SIDD and SIRD. MARD was characterised by a higher age at diabetes diagnosis; MOD by a high BMI; SIDD by a relatively low BMI, lower age and low insulin secretion (i.e. low HOMA-B); and SIRD by a high BMI and high HOMA-IR. MARD and MOD were additionally characterised by moderate metabolic derangement, and SIDD and SIRD by severe metabolic derangement [3]. We used the centroids and the same baseline variables to assign individuals to clusters.

### Variables

To characterise the population and evaluate its comparability with the population in the Ahlqvist et al study, we explored a broad range of additional characteristics. All variables are listed in electronic supplementary material (ESM) Table 1.

HbA_1c_ values were measured at baseline, and follow-up measurements were available from routine care through linkage with hospital data. The Short Form 36 (SF-36) questionnaire was completed at baseline and then once a year, with data currently available for 7 years of follow-up. The mental component summary (MCS) and physical component summary (PCS) scores were derived from the SF-36, which has been reported to be validated and reliable [18]. The SF-36 includes 36 questions contributing to eight health domains, which in turn contribute to MCS and PCS scores. These scores are calculated based on scoring all questions based on factor analysis, and transformed to a mean of 50 and an SD of 10, as described elsewhere [19].

### Outcomes

We used several outcomes in this study to characterise the different clusters, including complications at baseline, and HbA_1c_ and QoL during follow-up. The cluster-wise association with diabetes-related complications was determined at baseline, as follow-up data were not available for complications. The complications included CKD (defined as having an eGFR <60 ml/min per 1.73 m^2^ and/or albumin excretion of at least 30 mg/day), neuropathy (defined as the presence of neuropathic pain and/or impaired vibration sense), retinopathy (based on fundoscopy), non-alcoholic fatty liver disease (NAFLD) (defined as having at least 5.56% liver fat [20]), CVD and cerebrovascular disease. Cluster-wise first time to reach adequate glycaemic control was defined as an HbA_1c_ <53 mmol/mol (7.0%) during follow-up [21]. Finally, QoL was determined at baseline and during follow-up based on the MCS and PCS scores of the SF-36.

### Statistical analyses

All analyses were performed separately for individuals who were newly diagnosed with type 2 diabetes during their baseline visit to the Maastricht Study centre and for those who were already diagnosed with type 2 diabetes.

We used descriptive statistics to summarise cluster-wise and total baseline characteristics and compared these characteristics with χ^2^ tests for categorical variables and with one-way ANOVA for continuous variables.

Logistic regression models estimated ORs for the cluster-wise associations with the presence of diabetes-related outcomes and depression at baseline. These models were adjusted for age, sex, diabetes duration (only in the already diagnosed group) and educational level (with the ‘low’ category as the reference group). We performed two sensitivity analyses in which we replaced educational level by the International Socio-Economic Index of occupational status 2008 (ISEI-08) classification and by equivalent income, to see if these proxies of socioeconomic position had a different effect.

We depicted the evolution of HbA_1c_ over time by dividing the follow-up time into 6 month intervals, taking the mean of the measurements per interval per cluster and plotting these points over time.

A Kaplan–Meier curve was created to visualise the time to reach adequate glycaemic control (HbA_1c_ <53 mmol/mol [<7.0%]) and Cox proportional hazards models were used to estimate the HR of reaching adequate glycaemic control. These models were adjusted for age, sex, diabetes duration and educational level. We performed two sensitivity analyses in which we replaced educational level by the ISEI-08 classification and by equivalent income, to see if these proxies of socioeconomic position had a different effect. Moreover, we used Kaplan–Meier curves and Cox proportional hazards models to evaluate likely depression, defined as a deterioration of 3 points in MCS score. This proxy was used due to the absence of depression data during follow-up.

In our main analysis, we depicted QoL over time by plotting the yearly mean component scores (MCS and PCS) per cluster. Generalised estimating equations were used to adjust the plots for age and sex. We performed a sensitivity analysis by adding a correction for BMI to this model. In a separate sensitivity analysis we analysed the data by sex to evaluate whether there was a sex-specific effect (without adjustment for sex in this analysis).

An α level of 0.05 was used and data were analysed using IBM SPSS Statistics v.26 for Windows (IBM, Armonk, NY) and R language v.4.1 and RStudio v.1.4 (https://www.R-project.org/).

## Results

### Participant selection

**Fig. 1:**
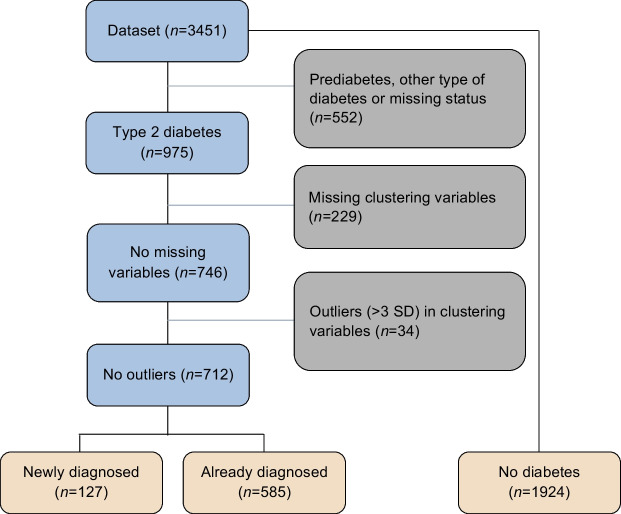
Flowchart of participant selection

Figure 1 shows the selection of participants from the initial dataset to sets of newly diagnosed individuals (*n*=127), already diagnosed individuals (*n*=585) with no missing values or outliers in the clustering variables and a population of people without diabetes (*n*=1924), used in the QoL analysis.

### Baseline characteristics

Tables 1 and 2 show the most important cluster-wise and total baseline characteristics of newly and already diagnosed individuals, respectively. Apart from the differences in clustering variables, there were significant differences in eGFR, MCS score, PCS score and number of people with neuropathy among newly diagnosed individuals. In already diagnosed individuals, there were significant differences in sex, diabetes duration, eGFR, PCS score, the number of people with retinopathy and the number of people using glucagon-like peptide-1 receptor agonists (GLP1-RAs), insulin and other glucose-lowering drugs. More characteristics of newly diagnosed and already diagnosed individuals are shown in ESM Tables 2 and 3, respectively, and characteristics of people without diabetes are shown in ESM Table 4.

**Table 1:** Total and cluster-wise baseline characteristics of newly diagnosed individuals

Characteristic	MARD (*N*=46)	MOD (*N*=33)	SIDD (*N*=21)	SIRD (*N*=27)	*p* value^a^	Total (*N*=127)	Missing
Age (years)							
At diagnosis	67.3±5.3	56.9±7.0	62.4±4.9	65.0±6.8	n/a	63.3±7.3	
At study visit	67.3±5.3	56.9±7.0	62.4±4.9	65.0±6.8	n/a	63.3±7.3	
Sex							
Men	25 (54.3)	21 (63.6)	17 (81.0)	18 (66.7)		81 (63.8)	
Women	21 (45.7)	12 (36.4)	<5 (19.0)	9 (33.3)	0.208	46 (36.2)	
BMI (kg/m^2^)	26.1±2.7	31.7±4.2	26.9±3.1	30.9±3.3	n/a	28.7±4.1	
Diabetes duration (years)	0.0±0.0	0.0±0.0	0.0±0.0	0.0±0.0	n/a	0.0±0.0	
HbA_1c_ (mmol/mol)	41.5±4.4	42.4±5.1	53.2±5.3	42.3±5.1	n/a	43.9±6.4	
HbA_1c_ (%)	5.9±0.4	6.0±0.5	7.0±0.5	6.0±0.5	n/a	6.2±0.6	
HOMA-B (%)	74.2±15.9	89.0±25.8	58.9±17.2	121.7±20.1	n/a	85.6±28.9	
HOMA-IR	1.8±0.5	2.3±0.6	2.0±0.8	3.2±0.6	n/a	2.2±0.8	
eGFR (ml/min per 1.73 m^2^)	85.3±16.6	93.0±13.3	88.0±14.9	79.8±14.1	0.009	86.6±15.5	1 (0.8)
SF−36							
MCS	54.3±6.8	50.1±13.1	57.0±4.1	55.1±5.8	0.022	53.9±8.7	<5 (3.1)
PCS	50.8±7.1	50.0±6.6	50.6±6.9	45.4±11.8	0.042	49.4±8.4	<5 (3.1)
History of							
CKD	9 (19.6)	<5 (9.1)	<5 (19.0)	6 (22.2)	0.528	22.0 (17.3)	
Neuropathy	<5 (4.3)	5 (15.2)	<5 (4.8)	8 (29.6)	0.010	16.0 (12.6)	
Retinopathy	0 (0.0)	0 (0.0)	0 (0.0)	0 (0.0)	n/a	0.0 (0.0)	14 (11.0)
CVD	12 (26.1)	6 (18.2)	6 (28.6)	10 (37.0)	0.434	34.0 (26.8)	

Data are mean±SD or *n* (%)

^a^No *p* values are specified for variables used in clustering, as the groups are separated based on these variables

n/a, not applicable

**Table 2:** Total and cluster-wise baseline characteristics of already diagnosed individuals

Characteristic	MARD (*N*=260)	MOD (*N*=103)	SIDD (*N*=94)	SIRD (*N*=128)	*p* value^a^	Total (*N*=585)	Missing
Age (years)							
At diagnosis	57.9±6.7	46.5±6.1	49.1±8.7	59.0±6.8	n/a	54.7±8.6	
At study visit	64.5±6.6	57.8±7.7	61.8±7.9	63.6±7.0	n/a	62.7±7.5	
Sex							
Men	186 (71.5)	63 (61.2)	74 (78.7)	82 (64.1)		405 (69.2)	
Women	74 (28.5)	40 (38.8)	20 (21.3)	46 (35.9)	0.025	180 (30.8)	
BMI (kg/m^2^)	27.0±3.1	33.2±4.0	29.6±3.8	32.5±4.3	n/a	29.7±4.5	
Diabetes duration (years)	6.5±4.9	11.3±7.2	12.8±8.3	4.5±3.6	<0.001	7.9±6.5	
HbA_1c_ (mmol/mol)	48.3±5.2	51.7±7.1	66.2±8.2	49.1±6.3	n/a	51.9±9.0	
HbA_1c_ (%)	6.6±0.5	6.9±0.6	8.2±0.8	6.6±0.6	n/a	6.9±0.8	
HOMA-B (%)	61.5±19.3	63.9±22.2	36.1±15.7	104.3±23.6	n/a	67.2±29.8	
HOMA-IR	1.7±0.5	2.0±0.7	1.9±0.9	3.3±0.7	n/a	2.1±0.9	
eGFR (ml/min per 1.73 m^2^)	85.1±15.4	89.4±18.7	87.5±20.1	77.3±16.0	<0.001	84.5±17.4	2 (0.3)
SF−36							
MCS	53.7±7.7	52.2±9.0	52.5±8.7	52.6±9.5	0.383	53.0±8.5	21 (3.6)
PCS	49.6±8.1	44.8±10.5	45.4±10.0	45.4±9.6	<0.001	47.2±9.4	21 (3.6)
History of							
CKD	55 (21.2)	36 (35.0)	25 (26.6)	33 (25.8)	0.058	149 (25.5)	
Neuropathy	55 (21.2)	23 (22.3)	24 (25.5)	34 (26.6)	0.626	136 (23.2)	
Retinopathy	9 (3.5)	6 (5.8)	9 (9.6)	<5 (1.6)	0.020	26 (4.4)	27 (4.6)
CVD	73 (28.1)	27 (26.2)	36 (38.3)	45 (35.2)	0.137	181 (30.9)	
Use of glucose-lowering medication							
Biguanides	219 (84.2)	85 (82.5)	76 (80.9)	101 (78.9)	0.613	481 (82.2)	
SUs	65 (25)	24 (23.3)	27 (28.7)	29 (22.7)	0.748	145 (24.8)	
TZD	<5 (1.5)	<5 (1.9)	0 (0)	0 (0)	0.281	6 (1)	
Alpha-glucosidase inhibitors	0 (0)	0 (0)	0 (0)	0 (0)	n/a	0 (0)	
DPP4-Is	15 (5.8)	6 (5.8)	7 (7.4)	10 (7.8)	0.848	38 (6.5)	
GLP1-RAs	0 (0)	<5 (1)	<5 (3.2)	<5 (0.8)	0.040	5 (0.9)	
SGLT2-Is	0 (0)	0 (0)	0 (0)	0 (0)	n/a	0 (0)	
Insulin	25 (9.6)	37 (35. 9)	56 (59.6)	<5 (3.1)	<0.001	122 (20.9)	
Other	0 (0)	<5 (1)	<5 (3.2)	<5 (0.8)	0.040	5 (0.9)	

Data are mean±SD or *n* (%)

^a^No *p* values are specified for variables used in clustering, as the groups are separated based on these variables

DPP4-I, dipeptidyl peptidase-4 inhibitor; n/a, not applicable; SGLT2-I, sodium-glucose cotransporter-2 inhibitor; SU, sulfonylurea; TZD, thiazolidinedione

### Complications at baseline

Table 3 shows the ORs for the presence of complications at baseline for newly diagnosed individuals. The SIRD cluster was associated with neuropathy. Overall, the numbers of complications were small in newly diagnosed individuals. Table 4 shows the ORs for the complications at baseline for already diagnosed individuals. The MOD cluster was associated with CKD (OR 3.04; 95% CI 1.62, 5.69). The SIDD cluster was associated with retinopathy, although this effect was no longer apparent after correction for education. The SIDD cluster was also associated with CVD, although this effect was no longer apparent after correction for diabetes duration. The presence of neuropathy, NAFLD and cerebrovascular disease did not differ between the clusters. The number of people with depression at baseline was too small to evaluate in newly diagnosed individuals. In already diagnosed individuals, the SIDD cluster was associated with depression (ESM Table 5).

**Table 3:** Cluster-wise ORs of complications at baseline for newly diagnosed individuals (*n*=127)

Variable^a,b^	*N* (%)	Unadjusted OR (95% CI)	Age/sex-adjusted OR (95% CI)	Age/sex/education category-adjusted OR (95% CI)^c^
CKD				
MARD	9 (19.6)	Reference		
MOD	<5 (9.1)	0.41 (0.10, 1.67)	1.29 (0.24, 6.81)	1.06 (0.19, 5.87)
SIDD	<5 (19.0)	0.94 (0.25, 3.49)	1.63 (0.37, 7.13)	1.17 (0.25, 5.53)
SIRD	6 (22.2)	1.14 (0.36, 3.66)	1.35 (0.39, 4.62)	1.21 (0.32, 4.53)
Neuropathy				
MARD	<5 (4.3)	Reference		
MOD	5 (15.2)	3.98 (0.72, 22.00)	7.91 (1.12, 55.89)	6.87 (0.95, 49.97)
SIDD	<5 (4.8)	1.08 (0.09, 12.56)	1.32 (0.10, 16.61)	1.13 (0.08, 15.10)
SIRD	8 (29.6)	1.76 (1.76, 46.71)	10.10 (1.89, 54.07)	8.72 (1.56, 48.69)
NAFLD				
MARD	10 (21.7)	Reference		
MOD	10 (30.3)	1.59 (0.57, 4.44)	1.08 (0.32, 3.65)	1.16 (0.34, 4.01)
SIDD	10 (47.6)	3.18 (1.05, 9.63)	2.46 (0.77, 7.87)	3.29 (0.94, 11.57)
SIRD	8 (29.6)	1.47 (0.50, 4.36)	1.30 (0.43, 3.94)	1.16 (0.37, 3.63)
CVD				
MARD	12 (26.1)	Reference		
MOD	6 (18.2)	0.64 (0.21, 1.92)	1.11 (0.30, 4.17)	1.12 (0.29, 4.30)
SIDD	6 (28.6)	1.10 (0.35, 3.49)	1.15 (0.33, 4.06)	1.13 (0.31, 4.18)
SIRD	10 (37.0)	1.62 (0.58, 4.50)	1.67 (0.56, 4.95)	1.77 (0.57, 5.45)
Cerebrovascular disease^d^				
MARD	<5 (4.3)	Reference		
MOD	0 (0.0)	n/a	n/a	n/a
SIDD	0 (0.0)	n/a	n/a	n/a
SIRD	<5 (1.6)	1.72 (0.23, 12.98)	1.70 (0.22, 13.36)	3.40 (0.33, 34.85)

^a^See Table 1 for the number of people in each cluster

^b^There were no newly diagnosed individuals with retinopathy (not shown)

^c^Using the ISEI-08 or equivalent income instead of education category did not alter the results

^d^Cerebrovascular disease was present only in the MARD and SIRD clusters

n/a, not applicable

**Table 4:** Cluster-wise ORs of complications at baseline for already diagnosed individuals (*n*=585)

Variable^a^	*N* (%)	Unadjusted OR (95% CI)	Age/sex-adjusted OR (95% CI)	Age/sex/education category-adjusted OR (95% CI)	Age/sex/education category/diabetes duration-adjusted OR (95% CI)^b^
CKD					
MARD	55 (21.2)	Reference			
MOD	36 (35.0)	2.01 (1.21, 3.33)	3.42 (1.95, 6.00)	3.42 (1.94, 6.02)	3.04 (1.62, 5.69)
SIDD	25 (26.6)	1.38 (0.80, 2.38)	1.57 (0.89, 2.78)	1.55 (0.87, 2.75)	1.39 (0.74, 2.60)
SIRD	33 (25.8)	1.30 (0.79, 2.14)	1.42 (0.86, 2.37)	1.40 (0.84, 2.33)	1.44 (0.86, 2.41)
Neuropathy					
MARD	55 (21.2)	Reference			
MOD	23 (22.3)	1.05 (0.61, 1.82)	1.46 (0.81, 2.62)	1.34 (0.74, 2.43)	1.36 (0.71, 2.61)
SIDD	24 (25.5)	1.20 (0.69, 2.10)	1.33 (0.75, 2.35)	1.22 (0.68, 2.17)	1.22 (0.65, 2.32)
SIRD	34 (26.6)	1.32 (0.81, 2.16)	1.40 (0.85, 2.30)	1.33 (0.80, 2.20)	1.33 (0.80, 2.20)
Retinopathy					
MARD	9 (3.5)	Reference			
MOD	6 (5.8)	1.74 (0.60, 5.02)	1.84 (0.60, 5.60)	1.49 (0.48, 4.65)	0.41 (0.11, 1.54)
SIDD	9 (9.6)	3.04 (1.17, 7.93)	2.86 (1.08, 7.54)	2.43 (0.91, 6.51)	0.61 (0.17, 2.16)
SIRD	<5 (1.6)	0.44 (0.09, 2.05)	0.45 (0.10, 2.14)	0.40 (0.08, 1.90)	0.52 (0.11, 1.01)
NAFLD					
MARD	71 (27.3)	Reference			
MOD	34 (33.0)	1.28 (0.78, 2.10)	1.02 (0.61, 1.73)	1.04 (0.61, 1.76)	1.13 (0.63, 2.02)
SIDD	29 (30.9)	1.18 (0.70, 1.98)	1.04 (0.61, 1.76)	1.07 (0.63, 1.83)	1.17 (0.65, 2.08)
SIRD	40 (31.3)	1.18 (0.75, 1.88)	1.18 (0.74, 1.88)	1.21 (0.75, 1.94)	1.19 (0.74, 1.91)
CVD					
MARD	73 (28.1)	Reference			
MOD	27 (26.2)	0.96 (0.57, 1.62)	1.17 (0.68, 2.02)	1.08 (0.62, 1.88)	1.05 (0.57, 1.92)
SIDD	36 (38.3)	1.71 (1.04, 2.82)	1.81 (1.09, 3.01)	1.68 (1.00, 2.82)	1.64 (0.93, 2.89)
SIRD	45 (35.2)	1.47 (0.93, 2.32)	1.53 (0.97, 2.42)	1.47 (0.92, 2.33)	1.48 (0.93, 2.36)
Cerebrovascular disease					
MARD	14 (5.4)	Reference			
MOD	5 (4.9)	0.89 (0.31, 2.54)	1.06 (0.35, 3.17)	1.11 (0.37, 3.36)	1.44 (0.42, 4.91)
SIDD	5 (5.3)	0.97 (0.34, 2.77)	1.05 (0.36, 3.02)	1.12 (0.83, 3.25)	1.40 (0.44, 4.43)
SIRD	7 (5.5)	1.00 (0.39, 2.55)	1.01 (0.40, 2.58)	1.06 (0.41, 2.72)	0.99 (0.39, 2.57)

^a^See Table 1 for the number of people in each cluster

^b^Using the ISEI-08 or equivalent income instead of education category did not alter the results

### HbA_1c_ during follow-up

**Fig. 2:**
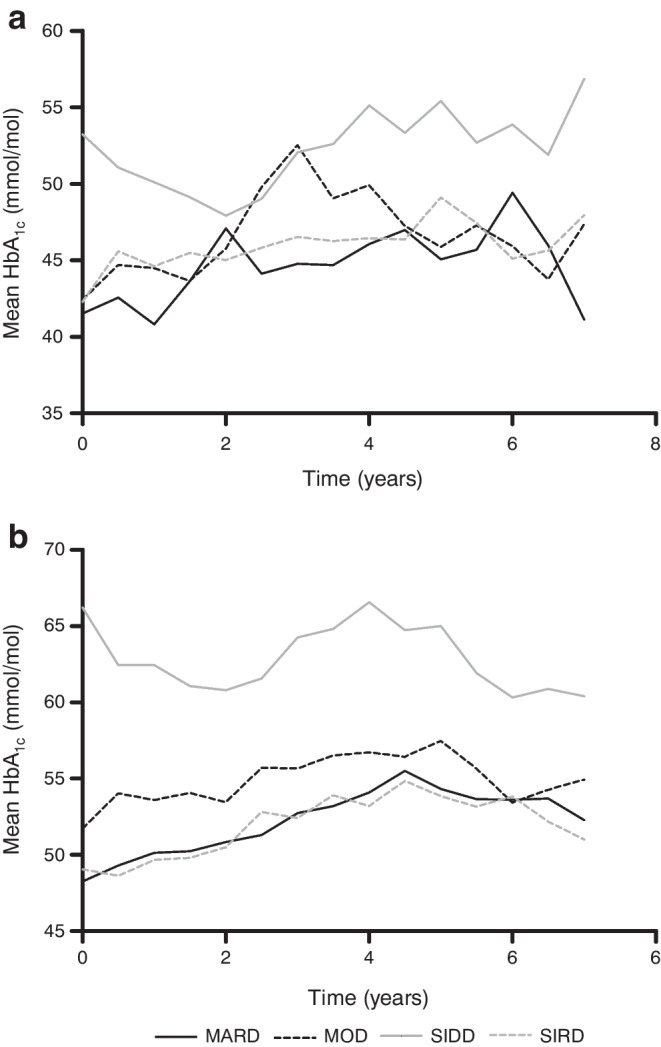
Cluster-wise evolution of HbA_1c_ (mmol/mol) during follow-up for newly diagnosed (**a**) and already diagnosed (**b**) individuals

**Fig. 3:**
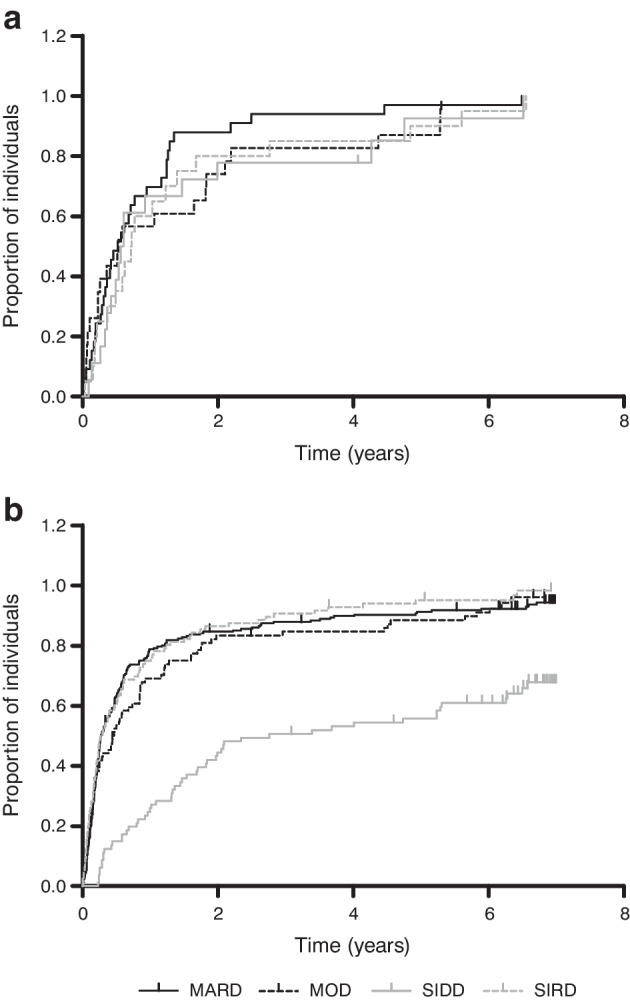
Kaplan–Meier curves of time to reach adequate glycaemic control (HbA_1c_ <53 mmol/mol [<7.0%]) for newly diagnosed (**a**) and already diagnosed (**b**) individuals

The analyses of HbA_1c_ during follow-up showed that already diagnosed individuals in the SIDD cluster were less likely to reach glycaemic control than individuals in the other clusters. Figure 2 shows the cluster-wise evolution of HbA_1c_ over time during the 7 years of follow-up. The mean HbA_1c_ of the SIDD cluster was consistently higher than that in the other clusters, in particular in the already diagnosed population. This is also reflected in the Kaplan–Meier curve of time to reach adequate glycaemic control (Fig. 3) and confirmed by the Cox regression, with an adjusted HR of reaching adequate glycaemic control of 0.31 (95% CI 0.22, 0.43) for the SIDD cluster compared with the MARD cluster. The Kaplan–Meier curve of newly diagnosed individuals (Fig. 3) did not indicate a difference in time to reach adequate glycaemic control between the clusters, and this was confirmed by the Cox regression (data not shown).

### QoL during follow-up

**Fig. 4:**
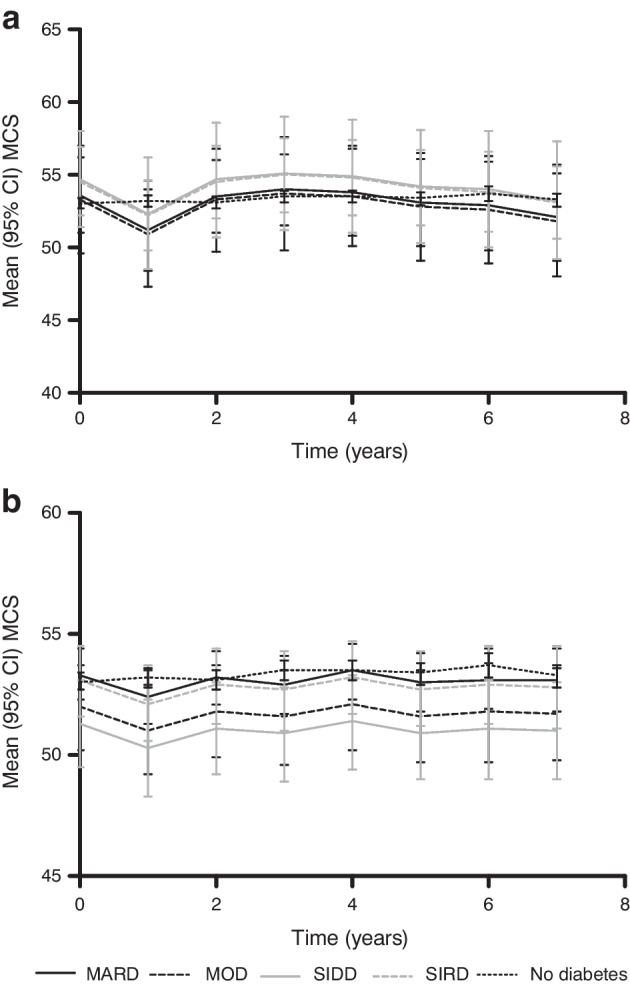
Cluster-wise evolution of MCS scores during follow-up for newly diagnosed (**a**) and already diagnosed (**b**) individuals compared with people without diabetes, adjusted for sex and age

**Fig. 5:**
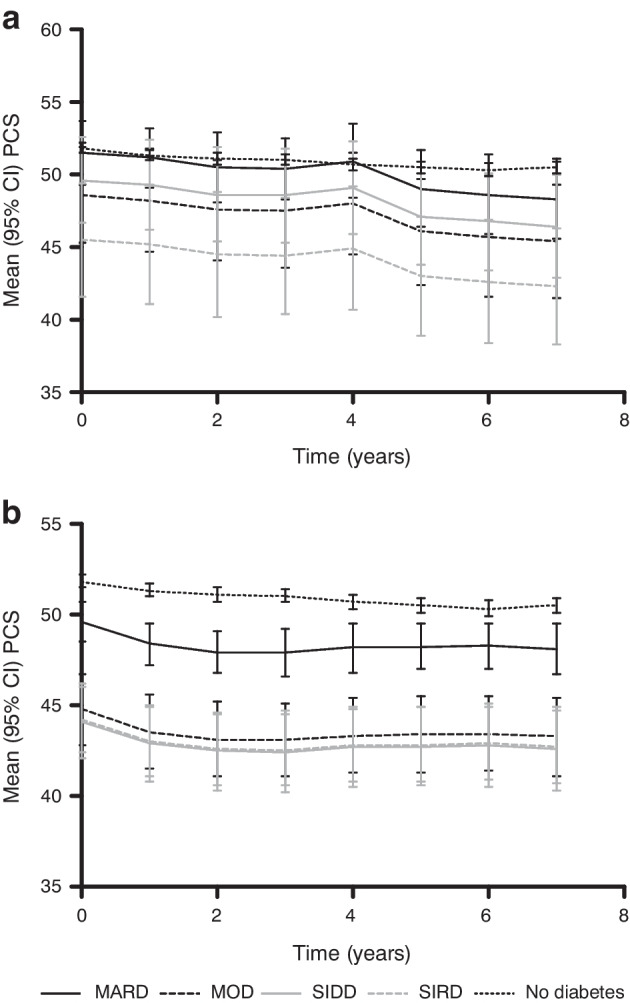
Cluster-wise evolution of PCS scores during follow-up for newly diagnosed (**a**) and already diagnosed (**b**) individuals compared with people without diabetes, adjusted for sex and age

Figures 4 and 5 show the evolution of QoL based on the SF-36 during the 7 years of follow-up. The mean MCS score (Fig. 4) appeared to fluctuate between 50 and 55 over time but was similar in all clusters and in people without diabetes overall. The mean PCS score (Fig. 5) seemed to decline slightly over time, with a decrease of approximately 3 points in newly diagnosed individuals and 1–2 points in already diagnosed individuals. The mean MCS score was lower in all already diagnosed clusters than in people without diabetes. Among already diagnosed people, the mean PCS score in the MARD cluster (approx. 48) was slightly lower than the score in people without diabetes (approx. 52), whereas the MOD, SIDD and SIRD clusters scored much lower (approx. 43). The differences in mean PCS score between clusters in newly diagnosed individuals were less obvious than those in already diagnosed individuals. People without diabetes scored highest, followed closely by people in the MARD cluster (mean PCS scores decreased from approx. 52 to 51 (no diabetes) and 50 (MARD) at 7 years), with the MOD and SIDD clusters having a slightly lower mean PCS scores over time (both mean PCS scores decreased from approx. 50 to 46 at 7 years). The SIRD cluster scored lowest, with the PCS score decreasing from around 45 to 43 at 7 years. Both MCS and PCS scores were lower in already diagnosed individuals than in newly diagnosed individuals.

The analysis in which we used a deterioration of at least 3 points in MCS score as a proxy for likely depression during follow-up showed no significant HRs (ESM Tables 6, 7), which was confirmed by the Kaplan–Meier curves (ESM Fig. 1).

### Sensitivity analyses

Using ISEI-08 or equivalent income instead of educational level for confounder correction did not change the results of the logistic regression or Cox proportional hazards model. Correcting QoL over time for BMI did not considerably change the resulting graphs. Analysing QoL by sex did not lead to differences in evolution of QoL.

## Discussion

The results of this study show that the terms ‘moderate’ and ‘severe’ used in the names of the novel clusters of type 2 diabetes are not reflected in individuals’ QoL. All individuals with diabetes scored lower than people without diabetes based on the PCS and MCS scores of the SF-36. The MOD cluster scored particularly low, although people in this cluster are labelled as having a ‘moderate’ degree of disease.

The use of the nearest centroid method led to four groups of people comparable to those reported by Ahlqvist et al [3]. Generally, the characteristics of the subgroups hold true in our population, with the differences between the clusters being smaller than those in the population studied by Ahlqvist et al. Their study population consisted of 14,652 Swedish adults with newly diagnosed diabetes. Compared with this population, our population was younger and showed less extreme values in HbA_1c_, HOMA-B and HOMA-IR.

Based on the Kaplan–Meier curves, our results match with those reported by Ahlqvist et al: the SIDD cluster was less likely to reach glycaemic control and thus appeared more therapy resistant than the other clusters. However, we only replicated this finding in already diagnosed individuals, not in newly diagnosed individuals. This is potentially due to the small number of people with newly diagnosed diabetes, or because of the limited metabolic derangement in this group to begin with.

Our results are also in line with studies reporting that diabetes is associated with a reduced QoL [14, 15], studies replicating the same clusters proposed by Ahlqvist et al by running a clustering algorithm [4–9] and those using the nearest centroid approach as we did [10, 11]. Similar to our findings, previous studies have shown that SIDD was the most therapy-resistant cluster [9] and SIRD was the cluster with the lowest physical fitness [10].

Some of the findings in previous studies were not confirmed in our study. First, there are reports of an increased risk of CKD in the SIRD cluster [3, 5, 6]. Although the absence of this association in our study could be due to population differences, it could also be due to the longer diabetes duration in the MOD cluster (11.3 years) than in the SIRD cluster (4.5 years). In addition, we did not replicate the typical evolution of HbA_1c_ after diabetes diagnosis, with a drop in HbA_1c_ after starting glucose-lowering treatment initially, but a subsequent slow increase in HbA_1c_ as treatment is no longer sufficient, as reported by Dennis et al [4]. This could be because baseline HbA_1c_ levels in newly diagnosed individuals were not markedly elevated. These individuals were diagnosed by chance (i.e. had no symptoms) during the visit to the study centre and probably did not seek treatment afterwards. The population in the study by Dennis et al was a trial population, with each individual starting on a glucose-lowering drug. Moreover, the 95% CIs of the cluster-wise associations with complications at baseline were wide. This indicates that the models had limited robustness, which could be due to the small number of people, in particular in the newly diagnosed group. The analysis of depression at baseline was also limited by the small number of events.

After confirmation that our clusters were comparable to those reported previously, we studied the cluster-wise evolution of QoL based on the MCS and PCS scores of the SF-36. People with type 2 diabetes had a lower QoL than those without, but the degree to which QoL was lower differed between the clusters. MOD, in particular, appeared to not be ‘moderate’ when looking at QoL, in particular PCS score. We hypothesised that this might be due to impaired functioning because of obesity, but adjustment for BMI turned out to have little effect. The question remains whether the observed differences in QoL scores are (clinically) relevant. This is a subjective matter, as there are no strict guidelines on the interpretation of MCS and PCS scores. Generally, a change of 2–3 points is considered to be relevant [19, 22]. This means that the observed differences in MCS and PCS scores can be interpreted on two levels: relevant difference (i.e. 2–3 points difference) and significant difference (i.e. no overlap in 95% CI).

For both already and newly diagnosed individuals, there were no significant or relevant differences in MCS scores between the clusters and people without diabetes. There were both significant and relevant differences in PCS scores among already diagnosed individuals between people without diabetes, MARD and the other three clusters (MOD, SIDD, SIRD). There were no significant differences in PCS scores among newly diagnosed individuals, but most of the differences were relevant. In newly diagnosed individuals, the MARD cluster scored relevantly lower in terms of PCS score than people without diabetes after approximately 5 years, whereas the other clusters scored relevantly lower from the beginning. All clusters, except for MOD and SIDD, scored relevantly differently from each other. In newly diagnosed individuals, PCS scores deteriorate over time and people in the SIRD cluster score lower than people in other clusters. In already diagnosed people, both the SIRD and SIDD clusters score lowest, followed by the MOD cluster. Assuming that Fig. 5a reflects PCS scores in the early stages of the disease and Fig. 5b reflects PCS scores in the late stages of the disease, it becomes evident that the SIRD cluster scores low from the beginning, whereas the SIDD and MOD clusters show a considerable decline in PCS score as diabetes progresses. People in the MARD cluster score higher than the other clusters, starting at a similar level to people without diabetes, but reach a relevantly different lower score after approximately 5 years. We also evaluated QoL by sex, but this did not lead to different results.

From the QoL graphs, it is clear that people with diabetes report lower QoL than people without diabetes, and that QoL differs between clusters of people with diabetes. In particular, the MARD cluster scored higher than the other clusters, whereas the MOD cluster scored similarly to the ‘severe’ clusters (SIDD and SIRD). Differences in MCS score were smaller than differences in PCS score. In line with these small differences in MCS, the HRs of likely depression based on the MCS score, as well as the Kaplan–Meier curves, showed no significant differences during follow-up.

Our results show that there is probably an effect of diabetes duration on QoL. The graphs show a decline in PCS score over time and the scores in newly diagnosed individuals are higher than in already diagnosed individuals. QoL might deteriorate as diabetes progresses, and this could also be an explanation for the limited findings in newly diagnosed individuals. People at the beginning of their disease appear to be more similar to each other and to people without diabetes, whereas differences become more apparent as the disease progresses.

This study has several strengths. The extensive exploration of the clusters allowed for confirmation of similar clusters to those reported previously, before moving on to exploration of QoL. The similarity of our clusters to those reported previously confirms the external validity of this study. The large set of variables allowed for extensive characterisation of the clusters. Finally, this is the first study, to our knowledge, to explore cluster-wise QoL. The availability of follow-up data on QoL allowed for investigation of its evolution over time, and the use of validated methods supports the validity of the study.

There are some limitations to keep in mind when interpreting our results. We included a relatively small sample size, leading to limited numbers of people in each cluster. In particular, the number of newly diagnosed individuals was small, meaning on the one hand that the results in this group should be interpreted with caution, but on the other hand that the non-significant findings could be significant in larger populations. Additionally, our population consisted of relatively ‘healthy’ people with type 2 diabetes and 99% of our population was white. It has been reported that different clusters might apply to other ethnicities [23–25]. Moreover, only self-reported sex was available and we could not account for different gender identities. As we did account for biological sex, we expect the absence of gender identities would have impacted the analysis of QoL only to a limited extent. We could not replicate all previous findings due to the data on comorbidities being available at baseline only. Finally, the study population might not be fully representative of the mental health of the general population, as the Maastricht Study includes only those willing to participate and visit the research centre; people with mental health problems may be less inclined to participate. This might have led to the small differences in MCS scores.

This study shows that the ‘moderate’ and ‘severe’ nomenclature of previously suggested clusters of type 2 diabetes is not entirely reflected in QoL. Of the clusters with diabetes, people in the MARD cluster scored relatively highly in terms of PCS score, whereas people in the MOD cluster reported lower PCS scores, comparable to those in the SIDD and SIRD clusters. This indicates that the MOD cluster is not ‘moderate’ in terms of impaired QoL, as it is similar to the ‘severe’ clusters. Although the ‘moderate’ and ‘severe’ annotations are reflected in the degree of metabolic derangement, they do not entirely hold up from an individual’s perspective in terms of QoL. It might be better to remove the ‘moderate’ and ‘severe’ annotations from the names of the clusters before implementing the clusters in practice, as this non-neutral nomenclature could affect disease perception of people with type 2 diabetes, their healthcare providers and society at large. Further research, preferably in larger populations, is required to confirm these findings and provide more support for reconsideration of the nomenclature used in clusters of type 2 diabetes.

### Supplementary Information

Below is the link to the electronic supplementary material.

Supplementary file1 (PDF 362 KB)

## Data Availability

The data of this study derive from the Maastricht Study, but restrictions apply to the availability of these data, which were used under licence for the current study. Data are, however, available from the authors upon reasonable request and with permission of the Maastricht Study management team.

## Abbreviations

CKD: Chronic kidney disease
GLP1-RA: Glucagon-like peptide-1 receptor agonist
ISEI-08: International Socio-Economic Index of occupational status 2008
MARD: Moderate age-related diabetes
MCS: Mental component summary
MOD: Moderate obesity-related diabetes
NAFLD: Non-alcoholic fatty liver disease
PCS: Physical component summary
QoL: Quality of life
SF-36: Short Form 36
SIDD: Severe insulin-deficient diabetes
SIRD: Severe insulin-resistant diabetes
