# Supplementary material for: Disease severity-based subgrouping of type 2 diabetes does not parallel differences in quality of life: the Maastricht Study
Source: Diabetologia. 2024 Jan 11;67(4):690–702. doi: 10.1007/s00125-023-06082-4 (PMC10904551; doi:10.1007/s00125-023-06082-4)
Supplement: Supplementary file 1 — Supplementary file1 (PDF 362 KB) [file 125_2023_6082_MOESM1_ESM.pdf]

ESM Table 1 List of variables used and how they were measured.

**Clustering variables (at baseline)**	
Age at diagnosis	Year of birth minus self-reported year of diabetes diagnosis.
Diabetes duration	Set to zero for newly diagnosed individuals, calculated based on self-reported year of diabetes diagnosis for already diagnosed individuals.
BMI	Calculated from height and weight measured at research centre.
HbA1c	Measured at research centre.
HOMA-B	Homoeostatic model assessment 2 of beta cell function. C-peptide levels used in calculation. Measured at research centre. Calculated with the HOMA calculator available from Calculated by use of the HOMA calculator available from https://www.dtu.ox.ac.uk.
HOMA-IR	Homoeostatic model assessment 2 of insulin resistance. C-peptide levels used in calculation. Measured at research centre. Calculated with the HOMA calculator available from Calculated by use of the HOMA calculator available from https://www.dtu.ox.ac.uk.
**Additional variables (at baseline)**	
Sex	Self-reported sex.
Caucasian ethnicity	Self-reported. White or others.
Smoking status	From basic smoking questionnaire at baseline.
Alcohol consumption	From Food Frequency Questionnaire at baseline
Waist circumference	Measured at research centre.
Healthy diet score	Based on adherence to the Dutch guidelines for a healthy diet Score between 0 (no adherence) and 100 (complete adherence) [1].
Total sedentary bouts	Per day. Mean value based on ActivPAL measurements for a week.
Mean sedentary time	Per day. Mean value based on ActivPAL measurements for a week.
MVPA	Moderate to vigorous physical activity.<br>Per day. Mean value based on ActivPAL measurements for a week.
Physical activity	Self-reported.
Wmax	Maximum power output at bicycle test, adjusted for body weight. Tertiles were created for age categories and sex into the low, average and high categories.
SBP	Systolic blood pressure. Measured at research centre.
DBP	Diastolic blood pressure. Measured at research centre.
HDL	High density lipoprotein. Measured at research centre.
LDL	Low density lipoprotein. Measured at research centre.
Total cholesterol	Measured at research centre.
Triglycerides	Measured at research centre.
Liver fat	Measured with Dixon Magnetic Resonance Imaging.
eGFR	Estimated glomerular filtration rate.<br>Based on CKD-EPI formula with serum creatinine and serum cystatin C measured at research centre.
Albumin excretion	Measured with 24 hr urine.
SF-36-MCS	Short form 36, mental component summary score.<br>SF-36 questionnaire completed at baseline.

SF-36-PCS	Short form 36, physical component summary score.<br>SF-36 questionnaire completed at baseline.
Presence of a current depressive episode based on the Mini International Neuropsychiatric Interview [2].	Presence of a current depressive episode based on the Mini International Neuropsychiatric Interview [2].
Mean self-reported health score (0=worst 100=best) from EQ5D	EQ_VAS from the EQ5D questionnaire. This question asks to rate ones health today from a scale of 0 (worst imaginable health) to 100 (best imaginable health).
Presence of retinopathy	Based on fundoscopy at research centre.
Neuropathy	Presence of neuropathic pain and/or impaired vibration sense based on neurothesiometer.
Health score	EQ5D questionnaire, score between 0(worst) and 100 (best).
Educational level	Questionnaire. Highest level of education. Low = No education, (un)completed primary education, or lower vocational education; Medium = Intermediate vocational education or higher secondary education; High = Higher vocational education or university education.
Income adjusted for household size	Questionnaire. Household income was divided by the square root of the household size.
Occupational status	Based on the International Socio-Economic Index 2008 (ISEI-08) of Occupational Status. Classified based on the relation between education and earnings, mediated by occupation.
Use of glucose-lowering medication	Participants were asked to bring their medication or a medication overview to the baseline visit. This provided the information on which glucose-lowering drugs were in use.
Microvascular disease	Defined as having retinopathy in one or both eyes, an eGFR <60ml/min/1.73m^2, albumin excretion ≥30mg/24 hour, or an impaired vibration sensation of one or both first toes.
Cardiovascular disease	Defined as having reported at least one of the following: myocardial infarction, cerebrovascular infarction and/or haemorrhage, percutaneous artery angioplasty of the coronary arteries, abdominal arteries, peripheral arteries or carotid artery, vascular surgery on coronary arteries, abdominal arteries, peripheral arteries or carotid artery.
Cerebrovascular disease	Cerebrovascular accident/stroke ever, from Rose Questionnaire [3].
**Follow-up variables**	
HbA1c	From routine medical care through linkage with hospital data.
SF-36-MCS	Short form 36, mental component summary score.<br>SF-36 questionnaire completed at yearly follow-up.
SF-36-PCS	Short form 36, physical component summary score.<br>SF-36 questionnaire completed at yearly follow-up.

All definitions are per the Maastricht Study's data dictionary

[1] Looman M, Feskens EJ, de Rijk M, Meijboom S, Biesbroek S, Temme EH, de Vries J, Geelen A. Development and evaluation of the Dutch Healthy Diet index 2015. Public Health Nutr. 2017 Sep;20(13):2289-2299.

[2] Sheehan DV, Lecrubier Y, Sheehan KH, et al. The Mini-International Neuropsychiatric Interview (M.I.N.I.): the development and validation of a structured diagnostic psychiatric interview for DSM-IV and ICD-10. J Clin Psychiatry. 1998;59 Suppl 20:22-57.

[3] Rose G, McCartney P, Reid DDSelf-administration of a questionnaire on chest pain and intermittent claudication.Journal of Epidemiology & Community Health 1977;31:42-48.

ESM table 2 Total and cluster-wise baseline characteristics of newly diagnosed individuals.

	MARD (N = 46)		MOD (N = 33)		SIDD (N = 21)		SIRD (N=27)		P-value[a]	Total (N = 127)		Missing	
Age at diagnosis (years)													
Mean (SD)	67.3	(5.3)	56.9	(7.0)	62.4	(4.9)	65.0	(6.8)	n/a	63.3	(7.3)		
<50	0	(0.0)	5	(15.2)	0	(0.0)	0	(0.0)		5	(3.9)		
50-59	7	(15.2)	14	(42.4)	5	(23.8)	5	(18.5)		31	(24.4)		
60-69	22	(47.8)	14	(42.4)	16	(76.2)	12	(44.4)		64	(50.4)		
≥70	17	(37.0)	0	(0.0)	0	(0.0)	10	(37.0)	n/a	27	(21.3)		
Sex													
Men	25	(54.3)	21	(63.6)	17	(81.0)	18	(66.7)		81	(63.8)		
Women	21	(45.7)	12	(36.4)	<5	(19.0)	9	(33.3)	0.208	46	(36.2)		
Caucasian ethnicity	46	(100.0)	33	(100.0)	20	(95.2)	27	(100.0)	0.165	126	(99.2)		
Smoking status													
Never	18	(39.1)	8	(24.2)	5	(23.8)	10	(37.0)		41	(32.3)		
Former (quit >6 months ago)	20	(43.5)	20	(60.6)	13	(61.9)	13	(48.1)		66	(52.0)		
Former (quit <6 months ago)	<5	(4.3)	0	(0.0)	<5	(4.8)	<5	(7.4)		5	(3.9)		
Current	6	(13.0)	<5	(9.1)	<5	(9.5)	<5	(7.4)		13	(10.2)		
Missing	0	(0.0)	<5	(6.1)	0	(0.0)	0	(0.0)	0.706	<5	(1.6)		
Alcohol consumption													
Mean (g/day) (SD)	17.9	(20.4)	19.0	(21.7)	12.9	(14.1)	12.0	(20.2)	0.442	16.1	(19.8)	5	(3.9)
None	6	(13.0)	7	(21.2)	5	(23.8)	8	(29.6)		26	(20.5)		
Low (women <=7. men <=14)	17	(37.0)	16	(48.5)	12	(57.1)	11	(40.7)		56	(44.1)		
High	22	(47.8)	9	(27.3)	<5	(19.0)	8	(29.6)		43	(33.9)		
Missing	<5	(2.2)	<5	(3.0)	0	(0.0)	0	(0.0)	0.200	<5	(1.6)		
Mean BMI (kg/m^2) (SD)	26.1	(2.7)	31.7	(4.2)	26.9	(3.1)	30.9	(3.3)	n/a	28.7	(4.1)		
Mean Waist circumference (cm) (SD)	95.1	(9.7)	106.2	(12.5)	100.7	(10.9)	106.7	(10.5)	0.000	101.4	(11.9)		
Dutch Healthy Diet (SD)	86.6	(15.1)	77.4	(12.7)	80.4	(15.2)	79.4	(12.5)	0.029	81.8	(14.4)	5	(3.9)

Mean total sedentary bouts in a week (SD)	336.7 (87.8)	308.5 (123.9)	355.9 (115.6)	285.7 (72.5)	0.163	322.2 (103.0)	32 (25.2)
Mean sedentary time (minutes/day) (SD)	573.4 (95.3)	564.1 (92.5)	582.2 (77.2)	584.5 (116.4)	0.892	574.3 (95.0)	32 (25.2)
Mean MVPA (minutes /day) (SD)	53.0 (25.9)	55.9 (29.4)	57.6 (21.9)	33.8 (16.6)	0.015	51.0 (26.0)	32 (25.2)
Estimated Wmax adjusted for body weight							
Mean (W/kg) (SD)	2.0 (0.6)	2.0 (0.5)	2.3 (0.6)	1.6 (0.5)	0.004	2.0 (0.6)	27 (21.3)
Low	11.0 (23.9)	15.0 (33.3)	<5 (23.8)	15.0 (55.6)		45.0 (35.4)	
Average	15.0 (32.6)	11.0 (6.1)	6.0 (28.6)	<5 (14.8)		36.0 (28.3)	
High	9.0 (19.6)	<5 (84.8)	7.0 (33.3)	<5 (3.7)		19.0 (15.0)	
Missing	11.0 (23.9)	5.0 (15.2)	<5 (19.0)	7.0 (25.9)	0.004	27.0 (21.3)	
Self-reported total physical activity (hours/week) (SD)	13.8 (6.9)	13.1 (7.3)	14.8 (8.3)	9.6 (6.3)	0.075	12.9 (7.3)	11 (8.7)
Mean diabetes duration (years) (SD)	0.0 (0.0)	0.0 (0.0)	0.0 (0.0)	0.0 (0.0)	n/a	0.0 (0.0)	
Mean HbA1c (mmol/mol) (SD)	41.5 (4.4)	42.4 (5.1)	53.2 (5.3)	42.3 (5.1)	n/a	43.9 (6.4)	
Mean HbA1c (%) (SD)	5.9 (0.4)	6.0 (0.5)	7.0 (0.5)	6.0 (0.5)	n/a	6.2 (0.6)	
Mean HOMA-B (%) (SD)	74.2 (15.9)	89.0 (25.8)	58.9 (17.2)	121.7 (20.1)	n/a	85.6 (28.9)	
Mean HOMA-IR (SD)	1.8 (0.5)	2.3 (0.6)	2.0 (0.8)	3.2 (0.6)	n/a	2.2 (0.8)	
Mean SBP (mmHg) (SD)	146.4 (21.7)	140.8 (17.0)	143.9 (18.9)	139.6 (16.1)	0.425	143.1 (19.0)	
Mean DBP (mmHg) (SD)	76.5 (11.0)	82.2 (9.3)	81.9 (10.5)	78.0 (8.6)	0.045	79.2 (10.2)	
Mean HDL (mmol/L) (SD)	1.5 (0.4)	1.2 (0.3)	1.3 (0.3)	1.3 (0.3)	0.027	1.3 (0.4)	
Mean LDL (mmol/L) (SD)	3.4 (1.2)	3.2 (1.0)	3.1 (1.4)	2.7 (1.0)	0.118	3.2 (1.2)	
Mean total cholesterol (mmol/L) (SD)	5.6 (1.3)	5.3 (1.0)	5.2 (1.5)	5.0 (1.1)	0.160	5.3 (1.3)	
Mean triglycerides (mmol/L) (SD)	1.8 (1.3)	1.9 (0.9)	2.0 (1.2)	2.2 (1.0)	0.494	1.9 (1.1)	
Mean liver fat (%) (SD)	6.7 (7.0)	8.8 (7.3)	9.6 (6.2)	9.4 (7.7)	0.836	8.3 (7.0)	51 (40.2)
Mean eGFR (ml/mn/1.73 m^2) (SD)	85.3 (16.6)	93.0 (13.3)	88.0 (14.9)	79.8 (14.1)	0.009	86.6 (15.5)	<5 (0.8)

Albumin excretion													
Mean (SD) (mg/24h)	17.0	(26.7)	15.3	(21.3)	12.9	(14.2)	16.0	(30.0)	0.937	15.7	(24.3)		
<30 mg/24h	41	(89.1)	30	(90.9)	18	(85.7)	23	(85.2)		112.0	(88.2)		
30-300 mg/24h	5	(10.9)	<5	(9.1)	<5	(14.3)	<5	(14.8)		15.0	(11.8)		
≥300 mg/24h	0	(0.0)	0	(0.0)	0	(0.0)	0	(0.0)		0.0	(0.0)		
Missing	0	(0.0)	0	(0.0)	0	(0.0)	0	(0.0)	0.889	0.0	(0.0)		
Mean self-reported health score (0=worst 100=best) from EQ5D (SD)	75.6	(21.1)	68.7	(23.2)	74.5	(24.1)	70.1	(28.4)	0.575	72.5	(23.7)	<5	(2.4)
SF-36													
Mean MCS (SD)	54.3	(6.8)	50.1	(13.1)	57.0	(4.1)	55.1	(5.8)	0.022	53.9	(8.7)	<5	(3.1)
Mean PCS (SD)	50.8	(7.1)	50.0	(6.6)	50.6	(6.9)	45.4	(11.8)	0.042	49.4	(8.4)	<5	(3.1)
History of													
Chronic kidney disease	9	(19.6)	<5	(9.1)	<5	(19.0)	6	(22.2)	0.528	22.0	(17.3)		
Neuropathy	<5	(4.3)	5	(15.2)	<5	(4.8)	8	(29.6)	0.010	16.0	(12.6)		
Retinopathy	0	(0.0)	0	(0.0)	0	(0.0)	0	(0.0)	-	0.0	(0.0)	14	(11.0)
NAFLD	10	(21.7)	10	(30.3)	10	(47.6)	8	(29.6)	0.203	38.0	(29.9)		
CVD	12	(26.1)	6	(18.2)	6	(28.6)	10	(37.0)	0.434	34.0	(26.8)		
Cerebrovascular disease	<5	(4.3)	0	(0.0)	0	(0.0)	<5	(7.4)	0.319	<5	(3.1)	<5	(0.8)
Use of glucose-lowering medication	N/A												
Educational level													
Low	21	(45.7)	10	(30.3)	<5	(9.5)	16	(59.3)		49.0000	(38.6)		
Medium	6	(13.0)	10	(30.3)	11	(52.4)	7	(25.9)		34.0000	(26.8)		
High	18	(39.1)	12	(36.4)	8	(38.1)	<5	(14.8)		42.0000	(33.1)		
Missing	<5	(2.2)	<5	(3.0)	0	(0.0)	0	(0.0)	0.002	<5	(1.6)		
Mean equivalent income (SD)	2033.3	(781.4)	1904.3	(770.4)	2068.6	(622.4)	1857.6	(925.7)	0.758	1966.3	(782.2)	24	(18.9)
Mean ISEI-08 classificaiton (SD)	52.2	(20.1)	49.8	(17.5)	52.5	(19.5)	48.3	(21.6)	0.874	50.8	(19.5)	30	(23.6)

Abbreviations: MARD moderate age-related diabetes, MOD moderate obesity-related diabetes, SIDD severe insulin-deficient diabetes, SIRD severe insulin-resistant diabetes, SD standard deviation, MVPA moderate to vigorous physical activity, HOMA-B homoeostatic model assessment 2 of beta cell function, HOMA-IR homoeostatic model

assessment 2 of insulin resistance, SBP systolic blood pressure, DBP diastolic blood pressure, NAFLD non-alcoholic fatty liver disease, ISEI-08 International Socio-Economic Index of occupational status 2008.

[a] no p-values are specified for variables used in clustering, since the groups are separated based on these variables.

ESM Table 3 Total and cluster-wise baseline characteristics of already diagnosed individuals.

	MARD(N =260)		MOD (N =103)		SIDD (N = 94)		SIRD (N=128)		P-value[a]	Total (N = 585)		Missing
Age at diagnosis (years)												
Mean (SD)	57.9	(6.7)	46.5	(6.1)	49.1	(8.7)	59.0	(6.8)	n/a	54.7	(8.6)	
<50	28	(10.8)	71	(68.9)	46	(48.9)	12	(9.4)		157	(26.8)	
50-59	126	(48. 5)	29	(28.2)	39	(41.5)	53	(41.4)		247	(42.2)	
60-69	94	(36.2)	<5	(2.9)	7	(7.4)	54	(42.2)		158	(27)	
≥70	12	(4.6)	0	(0)	<5	(2.1)	9	(7)	n/a	23	(3.9)	
Sex												
Men	486	(71.5)	63	(61.2)	74	(78.7)	82	(64.1)		405	(69.2)	
Women	74	(28.5)	40	(38.8)	20	(21.3)	46	(35.9)	0.025	180	(30.8)	
Caucasian ethnicity	254	(97.7)	99	(96.1)	89	(94.7)	127	(99.2)	0.180	569	(97.3)	
Smoking status												
Never	71	(27.3)	31	(30.1)	24	(25.5)	33	(25.8)		159	(27.2)	
Former (quit >6 months ago)	147	(56.5)	50	(48.5)	51	(54.3)	73	(57)		321	(54.9)	
Former (quit <6 months ago)	<5	(1.2)	<5	(3.9)	<5	(2.1)	<5	(0.8)		10	(1.7)	
Current	38	(14.6)	18	(17.5)	17	(18.1)	20	(15.6)		93	(15.9)	
Missing	<5	(0.4)	0	(0)	0	(0)	<5	(0.8)	0.713	<5	(0.3)	
Alcohol consumption												
Mean (g/day) (SD)	11.9	(15.2)	7.3	(13.0)	9.0	(12.0)	10.5	(13.4)	0.044	10.3	(14.0)	
None	61	(23.5)	41	(39.8)	24	(25.5)	44	(34.4)		170	(29.1)	
Low (women <=7; men <=14)	146	(56.2)	45	(43.7)	57	(60.6)	62	(48.4)		310	(53)	
High	53	(20.4)	17	(16.5)	13	(13.8)	22	(17.2)		105	(17.9)	
Missing	0	(0)	0	(0)	0	(0)	0	(0)	0.028	0	(0)	

Mean BMI (kg/m^2) (SD)	27.0 (3.1)	33.2 (4.0)	29.6 (3.8)	32.5 (4.3)	n/a	29.7 (4.5)	
Mean Waist circumference (cm) (SD)	99.3 (10.0)	113.0 (12.4)	107.0 (11.3)	111.5 (10.3)	<0.001	105.6 (12.3)	
Healthy diet (SD)	83.2 (14.1)	79.3 (15.2)	76.4 (13.5)	79.4 (12.6)	0.001	80.6 (14.1)	
Mean total sedentary bouts in a week (SD)	327.8 (107.9)	305.9 (91.1)	319.8 (134.3)	300.1 (83.3)	0.133	317.0 (105.8)	127 (21.7)
Mean sedentary time (minutes/day) (SD)	595.6 (96.5)	608.4 (123.0)	622.6 (103.1)	620.8 (84.3)	0.094	607.4 (100.4)	127 (21.7)
Mean MVPA (minutes /day) (SD)	47.6 (26.6)	40.5 (25.5)	39.9 (22.1)	36.5 (20.5)	0.001	42.8 (24.9)	127 (21.7)
Estimated Wmax adjusted for body weight							
Mean (W/kg) (SD)	2.0 (0.5)	1.8 (0.5)	1.8 (0.5)	1.6 (0.4)	<0.001	1.8 (0.5)	149 (25.5)
Low	78 (30.0)	52 (50.5)	36 (38.3)	75 (58.6)		241 (41.2)	
Average	93 (35.8)	18 (17.5)	23 (24.5)	15 (11.7)		149 (25.5)	
High	34 (13.1)	<5 (3.9)	<5 (2.1)	6 (4.7)		46 (7.9)	
Missing	55 (21.2)	74 (71.8)	33 (35.1)	32 (25.0)	<0.001	149 (25.5)	
Self-reported total physical activity (hours/week) (SD)	13.2 (7.3)	11.7 (7.8)	11.5 (8.6)	11.6 (7.1)	0.173	12.3 (7.6)	106 (18.1)
Mean diabetes duration (years) (SD)	6.5 (4.9)	11.3 (7.2)	12.8 (8.3)	4.5 (3.6)	<0.001	7.9 (6.5)	
Mean HbA1c (mmol/mol) (SD)	48.3 (5.2)	51.7 (7.1)	66.2 (8.2)	49.1 (6.3)	n/a	51.9 (9.0)	
Mean HbA1c (%) (SD)	6.6 (0.5)	6.9 (0.6)	8.2 (0.8)	6.6 (0.6)	n/a	6.9 (0.8)	
Mean HOMA-B (%) (SD)	61.5 (19.3)	63.9 (22.2)	36.1 (15.7)	104.3 (23.6)	n/a	67.2 (29.8)	
Mean HOMA-IR (SD)	1.7 (0.5)	2.0 (0.7)	1.9 (0.9)	3.3 (0.7)	n/a	2.1 (0.9)	
Mean SBP (mmHg) (SD)	142.4 (17.5)	141.5 (19.0)	145.1 (17.0)	140.1 (17.2)	0.199	142.2 (17.6)	
Mean DBP (mmHg) (SD)	76.3 (9.4)	78.6 (10.0)	76.4 (9.4)	77.4 (9.5)	0.169	77.0 (9.5)	
Mean HDL (mmol/L) (SD)	1.3 (0.4)	1.2 (0.3)	1.2 (0.4)	1.2 (0.3)	0.001	1.2 (0.4)	
Mean LDL (mmol/L) (SD)	2.4 (0.8)	2.2 (0.7)	2.2 (0.8)	2.3 (0.8)	0.140	2.3 (0.8)	
Mean total cholesterol (mmol/L) (SD)	4.3 (1.0)	4.2 (0.8)	4.2 (0.9)	4.4 (0.9)	0.136	4.3 (0 9)	

Mean triglycerides (mmol/L) (SD)	1.5 (0.7)	1.7 (0.7)	1.9 (1.2)	2.2 (1.1)	<0.001	1.7 (1.0)	
Mean liver fat (%) (SD)	7.2 (5.7)	10.1 (8.5)	8.2 (6.5)	10.1 (5.7)	0.005	8.4 (6.5)	274 (46.8)
Mean eGFR (ml/min/1.73 m^2) (SD)	85.1 (15.4)	89.4 (18.7)	87.5 (20.1)	77.3 (16.0)	<0.001	84.5 (17.4)	<5 (0.3)
Albumin excretion							
Mean (mg/24h) (SD)	23.2 (53.8)	46.3 (90.7)	51.6 (216.5)	23.1 (41.3)	0.044	31.7 (103.1)	7 (1.2)
<30 mg/24h	216 (83.1)	71 (68.9)	71 (75.5)	106 (82.8)		464 (79.3)	
30-300 mg/24h	41 (15.8)	25 (24.3)	19 (20.2)	20 (15.6)		105 (17.9)	
≥300 mg/24h	<5 (0.8)	<5 (3.9)	<5 (2.1)	<5 (0.8)		9 (1.5)	
Missing	<5 (0.4)	<5 (2.9)	<5 (2.1)		0.080	7 (1.2)	
Mean self-reported health score (0=worst 100=best) from EQ5D (SD)	75.3 (17.6)	69.6 (18.8)	67.5 (19.4)	70.7 (20.7)	0.002	72.1 (19.0)	18 (3.1)
SF-36							
Mean MCS (SD)	53.7 (7.7)	52.2 (9.0)	52.5 (8.7)	52.6 (9.5)	0.383	53.0 (8.5)	21 (3.6)
Mean PCS (SD)	49.6 (8.1)	44.8 (10.5)	45.4 (10.0)	45.4 (9.6)	<0.001	47.2 (9.4)	21 (3.6)
History of							
Chronic kidney disease	55 (21.2)	36 (35)	25 (26.6)	33 (25.8)	0.058	149 (25.5)	
Neuropathy	55 (21.2)	23 (22.3)	24 (25.5)	34 (26.6)	0.626	136 (23.2)	
Retinopathy	9 (3.5)	6 (5.8)	9 (9.6)	<5 (1.6)	0.020	26 (4.4)	27 (4.6)
NAFLD	71 (27.3)	34 (33)	29 (30.9)	40 (31.3)	0.692	174 (29.7)	
CVD	73 (28.1)	27 (26.2)	36 (38.3)	45 (35.2)	0.137	181 (30.9)	
Cerebrovascular disease	14 (5.4)	5 (4.9)	5 (5.3)	7 (5.5)	0.997	31 (5.3)	8 (1.4)
Use of glucose-lowering medication							
Biguanides	219 (84.2)	85 (82.5)	76 (80.9)	101 (78.9)	0.613	481 (85.2)	
SUs	65 (25)	24 (23.3)	27 (28.7)	29 (22.7)	0.748	145 (24.8)	
TZD	<5 (1.5)	<5 (1.9)	0 (0)	0 (0)	0.281	6 (1)	
Alfaglucosidase inhibitors	0 (0)	0 (0)	0 (0)	0 (0)	-	0 (0)	
DPP4-Is	15 (5.8)	6 (5.8)	7 (7.4)	10 (7.8)	0.848	38 (6.5)	
GLP1-RAs	0 (0)	<5 (1)	<5 (3.2)	<5 (0.8)	0.040	5 (0.9)	
SGLT2-Is	0 (0)	0 (0)	0 (0)	0 (0)	-	0 (0)	

Insulin	25	(9.6)	37	(35. 9)	56	(59.6)	<5	(3.1)	<0.001	122	(20.9)		
Other	0	(0)	<5	(1)	<5	(3.2)	<5	(0.8)	0.040	5	(0.9)		
Educational level													
Low	101	(38.8)	46	(44.7)	46	(48.9)	66	(51.6)		259	(44.3)		
Medium	75	(28.8)	32	(31.1)	23	(24.5)	30	(23.4)		160	(27.4)		
High	80	(30.8)	25	(24.3)	24	(25.5)	32	(25)		161	(27.5)		
Missing	<5	(1.5)	0	(0.0)	<5	(1.1)	0	(0.0)	0.298	5	(0.9)		
Mean equivalent income (SD)	1940.2	(803.8)	1756.9	(755.0)	1838.7	(710.9)	1830.1	(786.8)	0.337	1865.4	(777.7)	181	(30.9)
Mean ISEI-08 classification (SD)	49.9	(20.7)	48.0	(20.1)	47.6	(19.5)	48.4	(19.2)	0.794	48.8	(20.0)	472	(80.7)

Abbreviations: MARD moderate age-related diabetes, MOD moderate obesity-related diabetes, SIDD severe insulin-deficient diabetes, SIRD severe insulin-resistant diabetes, SD standard deviation, MVPA moderate to vigorous physical activity, HOMA-B homoeostatic model assessment 2 of beta cell function, HOMA-IR homoeostatic model assessment 2 of insulin resistance, SBP systolic blood pressure, DBP diastolic blood pressure, NAFLD non-alcoholic fatty liver disease, ISEI-08 International Socio-Economic Index of occupational status 2008.

[a] no p-values are specified for variables used in clustering, since the groups are separated based on these variables.

ESM Table 4 Characteristics of people without diabetes.

	Total (N = 1924)	Missing
Mean age (years) (SD)		
At study visit	57.9 (8.2)	
Sex		
Men	821 (42.7)	
Women	1103 (57.3)	
Mean BMI (kg/m^2)	25.5 (3.6)	<5 (0.1)
Mean HbA1c (mmol/mol) (SD)	36.1 (3.7)	7 (0.4)
Mean HbA1c (%) (SD)	5.5 (0.3)	7 (0.4)
Mean HOMA-B (%) (SD)	103.7 (26.0)	27 (1.4)
Mean HOMA-IR (SD)	1.3 (0.5)	27 (1.4)
Mean eGFR (SD) (ml/min/1.73 m^2)	90.2 (13.3)	21 (1.1)
SF-36		32 (1.7)
Mean MCS (SD)	51.7 (7.4)	32 (1.7)
Mean PCS (SD)	52.9 (8.2)	32 (1.7)
History of		
Chronic kidney disease	123 (6.4)	
Neuropathy	164 (8.5)	
Retinopathy	<5 (0.2)	178 (9.3)
CVD	248 (12.9)	

Abbreviations: SD standard deviation, HOMA-B homoeostatic model assessment 2 of beta cell function, HOMA-IR homoeostatic model assessment 2 of insulin resistance, eGFR estimated glomerular filtration rate, MCS mental component summary, PCS physical component summary, CVD cardiovascular disease.

ESM Table 5 Cluster-wise association with depression present at baseline in already diagnosed individuals.

	N (%)	Unadjusted OR (95%CI)	Age/sex adjusted OR (95%CI)	Age/sex/education category adjusted OR (95%CI)	Age/sex /education category/diabetes duration adjusted OR (95%CI)
MARD	10 (4.0)	reference	reference	reference	reference
MOD	7 (7.1)	1.80 (0.67-4.88)	1.56 (0.54-4.45)	1.57 (0.55-4.53)	1.35 (0.43-4.23)
SIDD	11 (12.2)	3.34 (1.37-8.17)	3.44 (1.38-8.56)	3.46 (1.38-8.69)	2.95 (1.05-8.27)
SIRD	9 (7.6)	1.94 (0.77-4.91)	1.80 (0.71-4.59)	1.81 (0.71-4.64)	1.87 (0.73-4.82)

Abbreviations: MARD moderate age-related diabetes, MOD moderate obesity-related diabetes, SIDD severe insulin-deficient diabetes, SIRD severe insulin-resistant diabetes, OR odds ratio, CI confidence interval.

ESM Table 6 Cluster-wise risk of having likely depression (change in MCS of at least -3) during 7 years of follow-up for newly diagnosed individuals.

	N (%)	Unadjusted HR (95%CI)	Age/sex adjusted HR (95%CI)	Age/sex/education category adjusted HR (95%CI)
MARD	22(47.8)	reference	reference	reference
MOD	33(48.5)	1.09(0.57-2.08)	1.31(0.60-2.84)	1.31(0.59-2.89)
SIDD	21(47.6)	0.92(0.43-1.94)	0.94(0.43-2.05)	0.95(0.42-2.14)
SIRD	27(40.7)	0.81(0.39-1.66)	0.82(0.39-1.71)	0.80(0.37-1.71)

Abbreviations: MARD moderate age-related diabetes, MOD moderate obesity-related diabetes, SIDD severe insulin-deficient diabetes, SIRD severe insulin-resistant diabetes, HR hazard ratio, CI confidence interval.

ESM Table 7 Cluster-wise risk of having likely depression (change in MCS of at least -3) during 7 years of follow-up for already diagnosed individuals.

	N (%)	Unadjusted HR (95%CI)	Age/sex adjusted HR (95%CI)	Age/sex/education category adjusted HR (95%CI)	Age/sex /education category/diabetes duration adjusted HR (95%CI)
MARD	109(41.9)	reference	reference	reference	reference
MOD	43(41.7)	0.99(0.69-1.41)	0.95(0.66-1.38)	0.98(0.67-1.42)	0.90(0.60-1.35)
SIDD	44(46.8)	1.13(0.79-1.60)	1.12(0.78-1.59)	1.13(0.79-1.62)	1.04(0.70-1.55)
SIRD	61(47.7)	1.18(0.86-1.62)	1.17(0.86-1.61)	1.18(0.86-1.62)	1.21(0.88-1.67)

Abbreviations: MARD moderate age-related diabetes, MOD moderate obesity-related diabetes, SIDD severe insulin-deficient diabetes, SIRD severe insulin-resistant diabetes, HR hazard ratio, CI confidence interval.

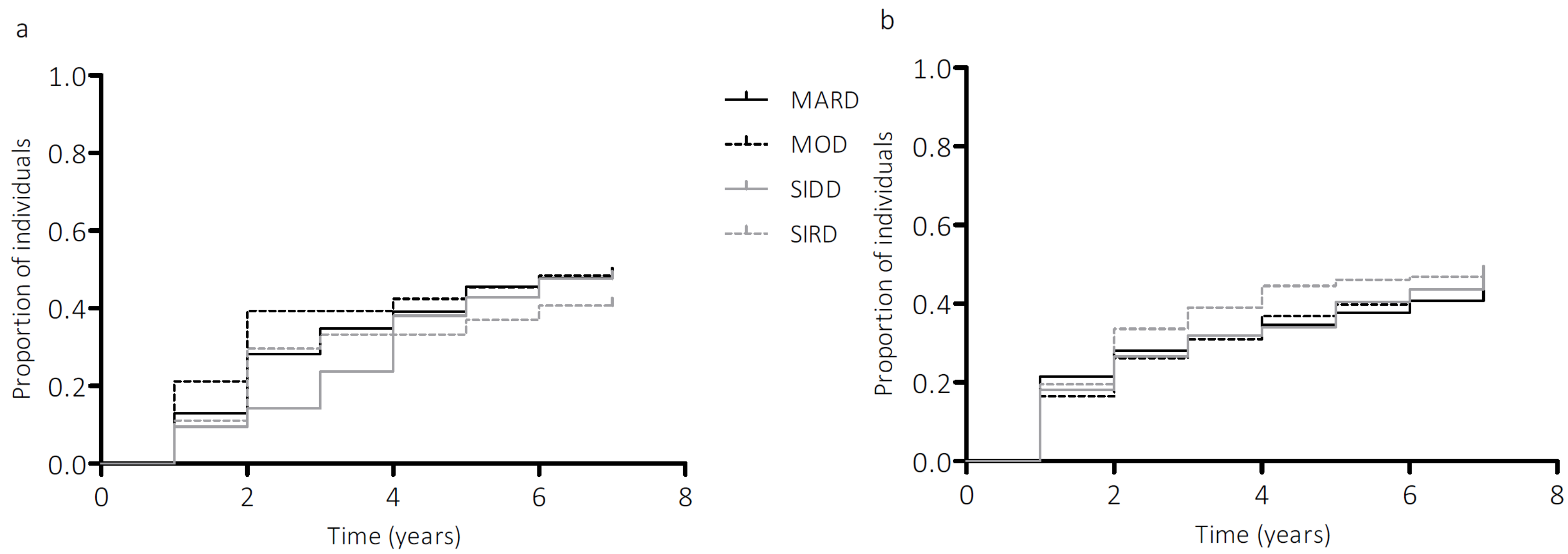

ESM Figure 1 Kaplan Meier of time to having likely depression (change in MCS of at least -3) for newly diagnosed individuals (a) and already diagnosed individuals (b).
